# Het succes van de buurtsportcoach: vertrouwen, maatwerk en continuïteit

**DOI:** 10.1007/s12508-021-00300-3

**Published:** 2021-07-09

**Authors:** André de Jeu, Marjon Stroet

**Affiliations:** Vereniging Sport en Gemeenten, Den Haag, Nederland

**Keywords:** buurtsportcoach, sportbeleid, gezondheidsbevordering, bewegen, Community sports motivator, Sports policy, Health promotion, Physical activity

## Abstract

In 2008, toen de combinatiefuncties werden ingevoerd, kon niemand nog bevroeden dat deze functie, tegenwoordig de buurtsportcoach genoemd, een groot succes zou worden. Anno 2021 doen vrijwel alle gemeenten in Nederland mee met wat inmiddels de Brede Regeling Combinatiefuncties heet. Zo heeft ook Sint Eustatius een buurtsportcoach. De waarde van de buursportcoach voor het gaan en blijven bewegen van mensen heeft zich inmiddels bewezen. De Vereniging Sport en Gemeente (VSG), nauw gelieerd aan de Vereniging Nederlandse Gemeenten (VNG), speelt vanaf het begin een actieve en belangrijke coördinerende rol bij het implementeren van de functie buurtsportcoach in Nederland. Belangrijke succesfactor van de gehanteerde strategie is: werken vanuit vertrouwen, maatwerk en continuïteit. In dit artikel wordt ingegaan op de verschillende regelingen rond de combinatiefuncties en de buurtsportcoach, de gehanteerde strategie om te komen waar we nu staan en perspectieven voor de nabije toekomst.

## Abstract (en)

In 2008, when the ‘combined functions’ (in Dutch ‘combinatiefuncties’) were introduced, no one could foresee the success of this position, now called the community sports motivators. In 2021, almost all Dutch municipalities participate in a broad program to stimulated physical activity (in Dutch ‘Brede Regeling Combinatiefuncties’). For example, in St. Eustatius there is a community sports motivator, too. The value of the community sports motivators – to stimulate and enhance physical activity – is evident. The Association of Sports and Municipalities (VSG), which is closely associated with the Association of Netherlands Municipalities (VNG), has played an active and important role in coordinating and implementing widely the community sports motivators in the Netherlands. Important success factors in the strategy to coordinate and implement the community sports motivators are mutual trust, tailoring and continuity. In this article an overview of the strategies used to implement the community sports motivators is provided, as well as some perspectives about the near future.

## De Impuls Brede scholen, Sport en Cultuur

Op 10 december 2007 ondertekenden de bewindslieden van de Ministeries van VWS en OCW, vertegenwoordigers van de Vereniging van Nederlandse Gemeenten (VNG), NOC*NSF, Verenigde Bijzondere Scholen (VBS) en de Cultuurformatie de bestuurlijke afspraken over de zogenaamde Impuls Brede scholen, Sport en Cultuur. Met de Impuls Brede scholen, Sport en Cultuur werden vier belangrijke doelstellingen nagestreefd [1, pag. 1]:

1. het uitbreiden van het aantal brede scholen met sport- en cultuuraanbod in zowel het primair als het voortgezet onderwijs;
2. het versterken van ongeveer 10 % van de sportverenigingen met het oog op hun maatschappelijke functie en het inzetten van sportverenigingen voor het onderwijs, de naschoolse opvang en de wijk;
3. het stimuleren van een dagelijks sport- en beweegaanbod op en rond scholen voor alle leerlingen;
4. bevorderen dat de jeugd tot 18 jaar vertrouwd raakt met een of meer kunst- en cultuurvormen, en het onder jongeren stimuleren van actieve kunstbeoefening.

In het eerste jaar werden gemeenten vanuit de Impuls Brede scholen, Sport en Cultuur volledig gefinancierd door het rijk voor het aanstellen van combinatiefunctionarissen. Deze combinatiefunctionarissen hadden als taak om sport of cultuur te combineren met ten minste één andere sector, zoals onderwijs, zorg, welzijn of het bedrijfsleven. Vanaf het tweede jaar moesten gemeenten het grootste deel (circa 60 %) zelf meebetalen. Voor de eerste fase, vanaf 2007, was het doel dat in 2012 2.500 combinatiefunctionarissen werkzaam waren in de sectoren onderwijs, sport en cultuur [1].

Vereniging Sport en Gemeenten (VSG) werd gevraagd de algemene en specifieke – op gemeenten gerichte – ondersteuning van de Impuls Brede scholen, Sport en Cultuur uit te voeren. Van VSG werd verwacht om naast de sportsector, ook de cultuur- en onderwijssector op zowel landelijk als regionaal niveau te ondersteunen met onder andere kennisoverdracht en kennisuitwisseling. Daarnaast waren er specifieke ondersteuning en advisering per sector georganiseerd. Zo deed NOC*NSF de advisering en ondersteuning vanuit de sector sport, de VBS het onderwijs en de Cultuurformatie de cultuursector. VSG is sinds 2007 de algemeen projectleider en ‘het gezicht’ van de combinatiefuncties.

Het huidige succes van de regeling is relatief eenvoudig tot stand gekomen. Vanaf het begin hebben de partijen samengewerkt vanuit onderling vertrouwen, hebben ze maatwerk geleverd en zijn ze steeds gericht geweest op continuïteit van de regeling. Dit resulteerde in een gestage groei van het aantal combinatiefunctionarissen en later, buurtsportcoaches. In 2008 zijn de 31 grootste gemeenten van ons land gestart met combinatiefuncties voor het primair of voortgezet onderwijs. Het grootste gedeelte richtte zich op de sportsector en een kleiner aantal op de cultuursector. Elk volgend jaar deden meer gemeenten mee. In de loop van de tijd zijn de landelijke regelingen veranderd, net als de focus. In 2021 beschikken vrijwel alle gemeenten over buurtsportcoaches (tab. 1).

**Table 1:** 

	looptijd	aantal te realiseren fte	focus
Impuls Brede Scholen, Sport en Cultuur	2008 tot en met 2011	2.250	jeugd tot 18 jaar
Brede Impuls Combinatiefuncties	2012 tot en met 2018	2.900	de buurt
Brede Regeling Combinatiefuncties	2019 tot en met 2022	3.665	inclusiviteit

## De combinatiefunctieregelingen

De opvolgende regelingen zijn door de tijd heen aangepast aan de actuele vragen in de sector (tab. 1). In 2012 is de regeling Impuls Brede Scholen, Sport en Cultuur uitgebreid naar alle leeftijdsgroepen, met een focus op bewegen en sporten in de buurt en daartoe omgedoopt tot Brede Impuls Combinatiefuncties. Sinds 1 januari 2019 is de Brede Regeling Combinatiefuncties (BRC) van kracht. Functionarissen die met termen als ‘buurtsportcoaches’, ‘combinatiefunctionarissen’ en ‘cultuurcoaches’ worden aangeduid (andere termen komen lokaal ook voor), werken aan het mogelijk maken van een leven lang inclusief sporten, bewegen en beoefenen van culturele activiteiten voor iedereen. Zoals afgesproken in de Bestuurlijke Afspraken BRC tussen de overheid en VNG doen zij dit onder andere door lokale verbindingen tussen verschillende sectoren tot stand te brengen en uit te bouwen, en door sport- en cultuuraanbieders duurzaam te versterken [2]. Hiermee zijn de combinatiefunctionarissen en de buurtsportcoaches een instrument dat in nagenoeg alle lokale/regionale sportakkoorden wordt ingezet om de beoogde doelen te realiseren.

Een belangrijke verandering van de BRC ten opzichte van de voorgaande regelingen is dat deze structureler wordt ingezet, omdat de rijksgelden telkens voor vier jaar worden toegekend in plaats van voor één jaar. Daarnaast staat het gemeenten vrij om in de BRC het normbedrag per fte (€ 50.000) als een richtlijn te hanteren. Dit betekent dat gemeenten naar eigen inzicht meer of minder fte kunnen inzetten dan het toegewezen aantal, en daarmee rekening kunnen houden met het gewenste opleidingsniveau van de combinatiefunctionaris. Tot slot heeft de BRC een bredere focus dan voorgaande regelingen, wat gemeenten de ruimte biedt om de functionarissen in te zetten zoals ze dat noodzakelijk achten. In 2019 nemen 347 van de 355 gemeenten deel aan de BRC, wat neerkomt op 98 procent van alle Nederlandse gemeenten. In de volgende paragrafen blikken we terug op de factoren die bijgedragen hebben aan dit succes.

## De situatie tot 2007: geen aandacht voor vertrouwen en maatwerk

Tot 2008, voordat de Impuls Brede scholen, Sport en Cultuur werd ingevoerd, waren er verschillende subsidies voor belangrijke projecten, zoals de Buurt Onderwijs en Sport-impuls, De Breedtesport Impuls, het Nationaal Actieplan Sport en Bewegen, en Meedoen Alle Jeugd door Sport. Kenmerkend voor deze projecten was dat gemeenten moesten voldoen aan de bijbehorende voorwaarden en criteria die waren opgesteld door de landelijke overheid, en daarop afgerekend werden. Wanneer gemeenten niet aan deze ‘blauwdruk’ voldeden, moesten ze het eerder verkregen subsidiegeld terugstorten. De blauwdruk had ook tot gevolg dat gemeenten in hun verantwoording naar het rijk niet konden opschrijven dat ze maatwerk hadden geboden. Het bieden van maatwerk betekent immers afwijken van de blauwdruk. In de praktijk werd natuurlijk wel maatwerk geboden. Dit betekende dus dat er geen inzicht was in het feitelijke resultaat en dat het geld mogelijk niet altijd op de bestemde plek terechtkwam. De ervaring leert dat het in de praktijk altijd even duurt voordat het geld van een regeling zo wordt benut als de bedoeling is. De genoemde projecten waren van te korte duur, waardoor projecten vaak al stopten voordat ze goed gingen lopen en er dus geen continuïteit geboden kon worden.

Medewerkers van de VNG, VWS directie sport, NOC*NSF en VSG hebben toen ingezet om meer te gaan werken vanuit vertrouwen. Dat paste ook in het tijdsbeeld. Het vierde kabinet Balkenende trad in 2007 aan en gebruikte het begrip ‘vertrouwen’ vaak. Gemeenten, als eerste en dichtstbijzijnde overheidsloket, hadden meer en meer de wens om maatwerk te (mogen) leveren voor hun burgers. Deze wens vormde ook een belangrijke basis voor de latere decentralisaties.

In het overleg tussen de driehoek van rijksoverheid, NOC*NSF en VNG/VSG vatte de opvatting post dat om écht het verschil te kunnen maken niet een zoveelste subsidie voor een project verstrekt moet worden, maar dat de verbinding tussen sport, cultuur en onderwijs mogelijk moet worden gemaakt op basis van de lokale behoeften, met de gemeente als coördinator en aanjager. In dit overleg werd het belang van vertrouwen benadrukt, omdat dat past bij het leveren van maatwerk op lokaal niveau. Dit betekende onder andere dat er vanuit de regeling zo min mogelijk regels voorgeschreven werden, dat er geen rode kaarten werden uitgedeeld als dingen anders liepen dan gepland, en dat gemeenten ook geen ‘strafkorting’ kregen, in de zin dat ze achteraf verkregen gelden moesten terugbetalen. Ook werden de regelingen structureel ingevoerd in plaats van projectmatig. Het gevolg van deze aanpak was dat het lokale enthousiasme voor deze combinatieregelingen groeide en dat de combinatieregelingen in de loop van de tijd werden uitgebreid naar alle leeftijdscategorieën.

## De inzet op continuïteit

De structurele inzet van de combinatieregelingen, en daarmee het bieden van continuïteit aan gemeenten, is achteraf gezien bijzonder, omdat Nederland in 2008 te maken kreeg met een economische recessie. Hoewel gemeenten het financieel vaak moeilijk hadden en de verschillende kabinetten als gevolg van de economische recessie fors moesten bezuinigen, bleef er voor de combinatiefuncties en later aanvullend voor de buurtsportcoaches geld bijkomen. Een belangrijke reden is dat verschillende onderzoeken steeds opnieuw hebben aangetoond dat de combinatiefunctionaris bijdraagt aan het bevorderen van de gezondheid. Het gaat bijvoorbeeld om het bevorderen van de participatie aan sport, de effecten van bewegen op schoolprestaties, de betekenis van sport en bewegen voor scholieren, complexe ketenvraagstukken in de jeugdzorg en het verkleinen van gezondheidsachterstanden.

Een tweede punt dat bijdroeg aan de continuïteit van de combinatiefunctieregeling is dat gemeenten na verloop van tijd wel de financiering moesten blijven organiseren, maar niet als enige de combinatiefunctionaris hoefden te cofinancieren. Dit betekent dat bijvoorbeeld een sportbedrijf of welzijnsorganisatie een buurtsportcoach kon inzetten omdat deze organisatie daar zelf voordeel van had. Toen daar bovenop de decentralisaties in de jeugdzorg en de Wmo plaatsvonden, ontdekte men in die beleidsvelden al snel de kansen die het inzetten van een buurtsportcoach biedt. De buurtsportcoach werd meer en meer een onderdeel van een ketenaanpak.

In de toen veel gebruikte zorgpiramide kwam het woord ‘sport’ in de nulde lijn, het voorliggende veld, merkwaardig nagenoeg niet voor. Tegelijkertijd besefte men wel steeds meer dat de ontwikkeling van de zorgkosten door participatie van datzelfde voorliggende veld teruggedrongen zou kunnen worden. Hoewel de strijd om het geld tussen enerzijds zorg en anderzijds preventie nog altijd op de klassieker Ajax-Feijenoord lijkt, is er steeds meer wetenschappelijk bewijs gekomen voor de social return on investment van sport en bewegen. Elke ingezette euro in sport en bewegen levert aantoonbaar een positief resultaat op in deelgebieden als arbeidsparticipatie, welbevinden (met lagere zorgvraag) en gezondheid [3].

## Dashboard SROI door sport

De noodzaak ontstond om meer inzicht te geven in de maatschappelijke waarde van sporten en bewegen. Het Kenniscentrum Sport en Bewegen heeft aan Rebel en het Mulier Instituut gevraagd de omvang van de SROI van sport en bewegen in Nederland te berekenen en de kosten en opbrengsten zo veel mogelijk in euro’s uit te drukken [3]. Deze SROI geeft de maatschappelijke kosten en opbrengsten weer die gerelateerd zijn aan mensen die sporten en bewegen, en de afweging van die kosten en opbrengsten.

Sinds 2020 is een SROI-dashboard beschikbaar waarmee de lokale inzet vergeleken kan worden met wat er landelijk gemiddeld wordt ingezet. Zowel de SROI, als bijvoorbeeld de beweegrichtlijn, de score op beweegvriendelijke omgeving en de gemeentelijke sportbegroting per inwoner wordt in het dashboard inzichtelijk gemaakt en er kan een scenario worden doorgerekend. Desgewenst kan een gemeente de uitkomsten van de doorrekening vergelijken met die van een groep vergelijkbare gemeenten. Met de resultaten kan de gemeente het eigen beleid op onderdelen aanpassen. De SROI is als instrument inzetbaar om van tevoren een voorspelling te doen over de uitkomst van de inzet van geld voor sport en bewegen. Voor het beleidsveld sport en bewegen vormt dit dashboard een stevige professionaliseringsslag.

## Naar een bredere inzet van de buurtsportcoach

De VNG en VSG hebben samen met de vele partners bewust ingezet op een brede ontwikkeling en versterking van de functie van de buurtsportcoach. Sinds 2008 zijn vele honderden bijeenkomsten georganiseerd, kleinschalig, dichtbij, lokaal en landelijk. Deze bijeenkomsten waren zowel gericht op de buurtsportcoaches zelf, als op de samenwerking met partners in andere beleidsvelden, zoals onderwijs, cultuur en zorg. Dit leidde tot verdere professionalisering van de buurtsportcoachfunctie, intersectoraal werken en werken op tactisch en strategisch niveau, en een differentiatie in het takenpakket van de buurtsportcoaches. Daar hoort ook een verruiming bij van de mogelijkheden tot verschillende salarisniveaus. Voor ongeveer de helft van de buurtsportcoaches is het bedrag in het rekenmodel van € 50.000 voldoende om de salariskosten te dekken. Voor de andere helft zijn de salariskosten gelijk aan het normbedrag of liggen de kosten daarboven. In de bestuurlijke afspraken van 2019 is ruimte gecreëerd om hier flexibel mee om te gaan.

Deze ontwikkelingen hebben bijgedragen aan een betere lokale beweeginfrastructuur, waarbij de buurtsportcoaches een cruciale, stimulerende en aanjagende rol spelen. Mede daardoor kunnen de lokale sportakkoorden een succes worden (349 gemeenten doen mee!). Die infrastructuur is goud waard. Het maakt het mogelijk om lokaal samen met allerlei partijen, dus niet alleen de sportsector, échte ketenaanpak na te streven en ook mensen te bereiken die kwetsbaar zijn en via de normale sportkanalen nauwelijks bereikt worden. Vroegsignalering is een begrip dat in de lokale en regionale akkoorden dan ook veelvuldig voorkomt. COVID-19 heeft ervoor gezorgd dat er veel meer oog is voor de kwetsbare burger en tevens het inzicht dat voldoende beweging en participatie aan sport en beweegactiviteiten voor iedereen van groot belang zijn.

## De nabije toekomst

De combinatiefunctieregeling heeft zichzelf in de loop van de jaren bewezen [4]. Dat wil niet zeggen dat er geen winst meer te behalen is. De samenwerking met partners uit het welzijnswerk, het bredere sociale domein, de publieke gezondheid en de zorg staan op lokaal niveau en zeker op landelijk niveau nog in de kinderschoenen. De wil is er, buurtsportcoaches zijn niet meer weg te denken, maar de doorontwikkeling vraagt om meer. De VNG en VSG zetten zich daarom namens de gemeenten in op meer buurtsportcoaches, die mede gefinancierd worden door andere ministeries of andere directies van VWS, dan de directie sport. De verwachting is dat het volgende kabinet nog meer gaat inzetten op preventie en vitaliteit, hetgeen vraagt om collectieve aanpakken. En daar kan de buurtsportcoach een belangrijke bijdrage aan leveren.

De zorgkosten lopen volgens de Volksgezondheid Toekomst Verkenning 2018 op naar 175 miljard in 2040 [5]. Met zijn allen worden we ouder en dat brengt meer chronische aandoeningen en bijbehorend (duur) medicijngebruik met zich mee. Vroegtijdig inzetten van bewegen met behulp van buurtsportcoaches kan deze ontwikkeling helpen beteugelen. Ook sportverenigingen hebben nog veel potentie om bij te dragen aan meer bewegen, vooral voor mensen die niet of nauwelijks sporten. Dat vraagt om het ontwikkelen van sportmogelijkheden op maat en een grotere inzet van vrijwilligers. Buurtsportcoaches kunnen daar een belangrijke ondersteunende rol bij spelen.

De invoering van de Gecombineerde Leefstijlinterventie, kortweg de GLI, in het basispakket van de zorgverzekering loopt moeizaam en kan beter. Er ligt een enorme kans om individuele of groepsgewijze begeleiding van gezonde leefstijl te verbinden met een buurtsportcoach-plus – een buurtsportcoach die gespecialiseerd is op alle onderdelen van de GLI én bewegen. In het ene geval uitvoerend, in het andere coördinerend. Naast lokaal maatwerk wordt daarmee ook individueel maatwerk mogelijk gemaakt. En dan hebben we het niet meer over Ajax-Feijenoord tussen de zorg en preventie. Voor de toekomst van de combinatiefunctieregeling zou dit een volgende logische stap zijn.
